# Dynamical Principles of Two-Component Genetic Oscillators 

**DOI:** 10.1371/journal.pcbi.0020030

**Published:** 2006-03-31

**Authors:** Raúl Guantes, Juan F Poyatos

**Affiliations:** 1 Instituto Nicolás Cabrera, Facultad de Ciencias C–XVI, Universidad Autónoma de Madrid, Madrid, Spain; 2 Evolutionary Systems Biology Initiative, Structural and Computational Biology Programme, Spanish National Cancer Centre (CNIO), Madrid, Spain; University of Tokyo, Japan

## Abstract

Genetic oscillators based on the interaction of a small set of molecular components have been shown to be involved in the regulation of the cell cycle, the circadian rhythms, or the response of several signaling pathways. Uncovering the functional properties of such oscillators then becomes important for the understanding of these cellular processes and for the characterization of fundamental properties of more complex clocks. Here, we show how the dynamics of a minimal two-component oscillator is drastically affected by its genetic implementation. We consider a repressor and activator element combined in a simple logical motif. While activation is always exerted at the transcriptional level, repression is alternatively operating at the transcriptional (Design I) or post-translational (Design II) level. These designs display differences on basic oscillatory features and on their behavior with respect to molecular noise or entrainment by periodic signals. In particular, Design I induces oscillations with large activator amplitudes and arbitrarily small frequencies, and acts as an “integrator” of external stimuli, while Design II shows emergence of oscillations with finite, and less variable, frequencies and smaller amplitudes, and detects better frequency-encoded signals (“resonator”). Similar types of stimulus response are observed in neurons, and thus this work enables us to connect very different biological contexts. These dynamical principles are relevant for the characterization of the physiological roles of simple oscillator motifs, the understanding of core machineries of complex clocks, and the bio-engineering of synthetic oscillatory circuits.

## Synopsis

Periodic variations in protein abundances are at the heart of important cellular processes, with common examples being the circadian rhythms or the cell cycle. What is the molecular basis of this behavior? Recent reports showed how simple architectures, based on the interaction of a few molecular components, are capable of inducing oscillatory dynamics. These structures are also of interest for the understanding of complex cellular oscillators, as they appear to be core constituents of them. The authors carefully analyze one of these architectures and uncover how different genetic implementations of such structures strikingly influence its dynamical behavior. They consider two genetic implementations of a repressor and activator element combined in a simple logical motif. While activation is transcriptionally implemented in both cases, repression may act either transcriptionally or post-translationally. These differences in design originate drastic changes in the way oscillations are produced, in the tolerance to molecular noise, or in the circuit response to external stimuli. Similar aspects have been discussed in relation to neural dynamics; therefore this work is able to connect two very different biological scenarios. Thus, simple genetic motifs exploit not only their connectivity pattern but their design to act as information processing units within living cells.

## Introduction

Oscillations play a fundamental role in many aspects of cell physiology. This is the case, for instance, of the well-known sustained oscillations associated to circadian clocks, enzyme synthesis, or the cell cycle [1]. In many of these situations, oscillations are originated by the interaction of many components forming complex regulatory networks, whose main constituents are being experimentally determined.

Recently, simple oscillator architectures have been found to be involved in the regulation of seemingly unrelated biological processes. An oscillator based on a combination of positive and negative feedback loops has been shown to regulate the cell cycle of Xenopus laevis embryos [2]. The combination of a two-component negative feedback between Cdc2 and the anaphase-promoting complex, with a positive feedback centered on Cdc2, leads to robust oscillations. The tumor suppressor protein p53, one of the most extensively studied proteins in relation to cancer [3], also seems to be part of a genetic oscillator [4]. Single cell experiments uncovered the existence of oscillations originated by the interactions of two components, p53 itself and Mdm2—one of its major regulators. While this simple scheme only originates damped oscillations, the presence of a putative positive feedback on p53 or delays in protein production could promote undamped behavior. These findings suggested a digital, instead of analogical, action of the p53-Mdm2 system, which could function as a fail-safe mechanism to maintain low p53 levels under general physiological conditions. A third system was uncovered in the context of somite segmentation [5] and linked to the oscillatory production of the Notch effector protein Hes1 [6]. These oscillations seem to be regulated by the presence of delays in a single negative feedback loop constituted by the very same protein [7,8], although a three-component negative feedback loop has been postulated [6]. Finally, a genetic clock was discovered in relation to the nuclear factor kappa B (NF-κB) [9], a transcription factor that regulates several cellular responses [10]. Oscillations, in this case, were associated with the interactions of proteins of the IκB family, which act as inhibitors of NF-κB. Three different isoforms of this inhibitor (IκBα, -β, and -ɛ) were revealed to contribute in distinct ways to the “decoding” of external information affecting the system. These same oscillations were later studied using time-lapse single-cell analysis [11]. As a consequence, the NF-κB oscillator is proposed to act as a complex control module able to use period or amplitude to differentially regulate expression of target genes. Overall, these studies indicate the relevance of broadly characterizing the dynamical properties of minimal motifs.

Full understanding of the oscillatory dynamics of simple schemes is also relevant for the development of synthetic gene networks. Artificial networks can be constructed following the suggestions derived from mathematical modeling and then characterized by combining theoretical and experimental studies [12]. This methodology has already been successfully applied in the case of oscillatory genetic networks. A bacterial oscillator was developed with the use of three well-known repressors linked in a daisy chain [13]. More recently, a combination of a positive and negative feedback constituting a two-component clock was both theoretically and experimentally characterized in Escherichia coli [14]. These two systems illustrate some of the advantages of studying basic properties of cellular oscillations in a context-independent biological setting.

Finally, the study of simple architectures can also help us identify common oscillator properties and their contribution to the dynamics and the modular assembly of more complex networks [15–18].

**Figure 1:**
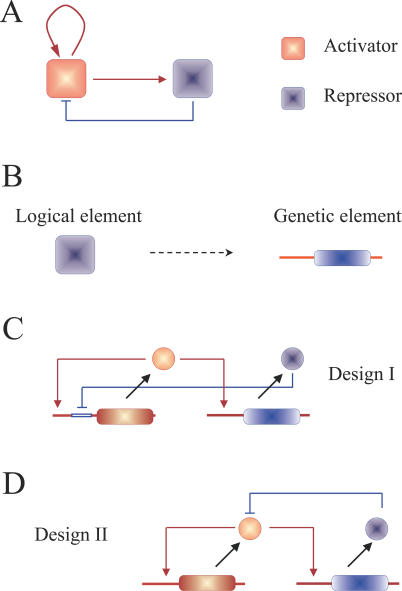
Minimal Oscillatory Architecture and Its Genetic Implementation (A) An activator (red) is acting on itself and on a repressor element (blue). The repressor is in turn acting on the activator. (B) The logical elements correspond to the promoter and coding region of a given gene. This motif can be genetically implemented in two ways. An activator protein operates transcriptionally in both cases while repression is implemented at the transcriptional, Design I (C), or post-translational, Design II (D) level.

In this work, we focus on understanding the interplay between genetic design and the functional properties of one of these minimal logical architectures. In particular, we consider a relaxation-based oscillator combining an activator and a repressor unit operating on each other (Figure 1A and 1B). In order to be able to exhibit sustained oscillations, this system also requires an autocatalytic step [19]. Thus, the activator is acting both on the repressor and on itself. This is not only a useful architecture to understand information processing of simple oscillators but also appears as a common core motif in unrelated biological contexts, such as the previously mentioned embryonic cell-cycle oscillator or the circadian clocks [2,17,18]. We introduce two different genetic implementations of this scheme. Both designs largely differ in the way the onset of oscillations is produced. These differences have major implications in the oscillatory behavior, stimulus response, robustness to biochemical noise, and synchronization by a periodic signal. The contrast between both implementations should be more stressed for networks operating close to the bifurcation regime, e.g., oscillatory responses, although some of the main features persist for a broad parameter range. A prominent class of biological relaxation oscillators, where this difference in dynamics is crucial, is neurons. Neural computational properties drastically depend on the way oscillations are generated. Following these dynamical principles, neurons are generally classified as Type I or Type II [20,21]. We are able to import some of these concepts into a completely different biological scenario.

## Results

### Models

We introduce two designs with repression operating differently. In Design I (Figure 1C), the repressor inhibits transcription of the activator in a sigmoidal way, e.g., we consider that the repressor binds to DNA as homodimer. This is a frequent biological situation [22], and it was recently used in the construction of a synthetic genetic clock [14]. In Design II (Figure 1D), the repressor antagonizes activator action, e.g., it acts as a protease increasing the activator degradation linearly. This design is related to previous theoretical studies of relaxation-based genetic oscillators, which were considered as valid core mechanisms of circadian clocks [16,17].We can mathematically describe the deterministic dynamics of the two implementations by means of a set of differential equations. The corresponding full models describe the dynamics of the biochemical reactions associated with the activator and repressor elements, i.e., transcription, translation, promoter binding, etc. We can simplify these models by using standard quasi steady-state assumptions.

Design I:


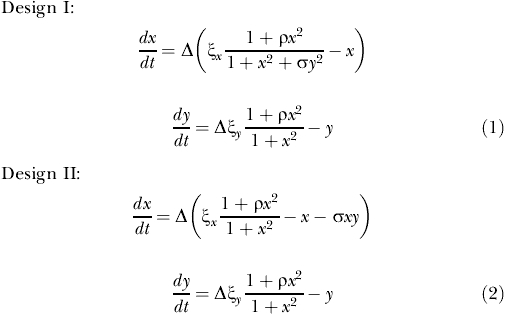


Here, *x,y* denote the activator (repressor) concentrations, Δ is the ratio of degradation rates between activator and repressor, σ is the repressor strength, ρ the increase of protein production due to the binding of the activator to the promoter, and ξ_*x*_(ξ_*y*_≡ɛξ_*x*_) is the effective activator (repressor) basal rate with ɛ measuring the ratio between them. Time and protein concentration are expressed in non-dimensional form (see Supporting Information for details on the models, simplifying assumptions, and non-dimensionalization of variables).

### Onset of Oscillations

When would these systems exhibit sustained oscillations? Both implementations are examples of relaxation-based oscillators. Oscillations appear when a clear separation of time scales between activator and repressor dynamics exists. This implies two conditions on the parameters: firstly, activator degradation should be stronger than the repressor one (Δ≫1), and secondly the activator translation rate should be also stronger than the repressor one (ɛ≪1, i.e., ξ_*y*_≪ξ_*x*_). The above conditions imply much faster activator dynamics, which “relaxes” the “stress” accumulated during the slow evolution of the repressor [23].

**Figure 2:**
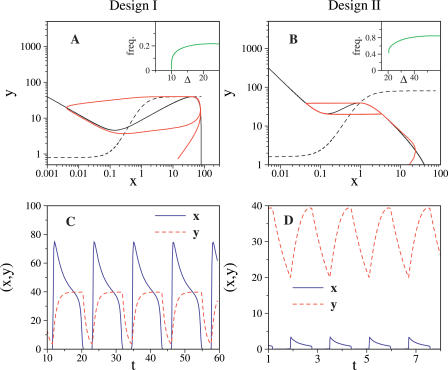
Repressor and Activator Dynamics (A and B) Shows repressor *(y)*, activator *(x)* phase plane analysis for both designs. Nullclines and limit cycle trajectory (red line) close to the bifurcation point. Solid line, activator-nullcline; dashed line, repressor-nullcline; inset, frequency of the limit cycle oscillations as a function of the bifurcation parameter Δ. Note the difference in both designs (oscillations may arise almost with zero frequency in Design I). (C and D) Activator (*x*, solid blue line) and repressor (*y*, dashed red line) adimensional concentration as a function of time. We consider the following parameter values in all figures: ξ_x_ = 1.58, ɛ = 0.05, *ρ* = 50, σ = 1 (see Supporting Information for bifurcation diagrams associated with the emergence of oscillations).

Although both implementations share a common structure capable to generate oscillations, their dynamical behavior is drastically different. This is a direct consequence of how repression acts on each system. This difference can be understood by analyzing the associated response curves (nullclines) in the phase plane. These curves depict the equilibrium concentration of one species as a function of the other one [23]. Sigmoidal repression in Design I permits the coexistence of high levels of the oscillator molecular constituents. High repressor concentration is required to actively shut off transcription of the activator. Thus, higher concentrations of both proteins are reached. This is reflected in the geometry of both nullclines which can intersect three times, i.e., there exists three equilibrium points, two of which have high concentration of both proteins (Figure 2A). In comparison, the linear repression in Design II implies a single equilibrium point at lower concentrations (Figure 2B). These differences are thus a consequence of the faster repressor action in the post-translational design with respect to the transcriptional one.

The different types of equilibria found in the two designs and their stability properties completely determine the onset of oscillations. In a situation without oscillations, Design I shows two equilibria, one stable and one unstable, with high concentration of both proteins. A change in a system parameter can cause these equilibria to approach each other until they coalesce and disappear, giving rise to a stable limit cycle causing the oscillations (saddle-node bifurcation on an invariant circle, Figure S1). In Design II, the single equilibrium point, stable when the system is not oscillating, may become unstable as a parameter changes and the system approaches a stable limit cycle (subcritical Hopf bifurcation, Figure S1).

### Oscillatory Features

To highlight the distinct features associated with genetic design, we considered similar biochemical parameters for both implementations. This implies equal transcription, translation, and binding rates. We also selected repression strength, σ, in such a way that mRNAs and repressor species show similar concentration levels in both systems. The most apparent property distinguishing both designs is the difference in period and amplitude of the oscillations. We characterized the shape of the oscillations in both cases as a function of the ratio of degradation rates, i.e., Δ, as this is one of the key parameters responsible for the different time scales between activator and repressor dynamics.

In Design I, after the saddle-node bifurcation, the period of the limit cycle can be arbitrarily large and changes appreciably with Δ. This is shown in the right inset of Figure 2A, where the frequency of the oscillations is plotted as a function of Δ. Oscillations might emerge at almost zero frequency. In addition, the relatively long time necessary to achieve strong repression allows the presence of large concentration amplitudes in this system (Figure 2C). Design II oscillations appear, however, with finite frequency and this tends to be a less variable characteristic of the system. Faster (linear) repression action prevents (in this case) large activator concentrations (Figure 2D).

The previous features can be qualitatively understood by inspection of the response curves in a regime where the parameter Δ is close to its bifurcation value, i.e., the value where oscillations arise. Large periods in Design I appear as a consequence of a slowdown in the dynamics of the activator after reaching its maximal amplitude. This corresponds to the point in phase space where the activator and repressor response curves are tangent. These slow dynamics are a remnant or ghost of the presence of a previous stable equilibrium [23]. Note that since the equilibrium is in the saturated part of the repressor-response curve, small changes in the activator (*x*) do not affect the equilibrium value of the repressor (*y*). This translates into oscillations with a broad “shoulder” of high activator and repressor concentrations (Figure 2C) and consequently into large periods. On the other hand, the equilibrium point in Design II changes stability when the maximum of the activator nullcline crosses the repressor curve below its saturated regime. Therefore, dynamics at these concentration levels can not be arbitrarily delayed (Figure 2D).

A further noticeable difference in oscillatory behavior is the appearance of damped oscillations associated to subthreshold values of Δ. This is seen only in the second design due to presence of a low-amplitude unstable limit cycle characteristic of subcritical Hopf bifurcations (Figure S1). Damped oscillations can emerge as a response to subthreshold stimuli and also play a role in aspects such as noise resistance or entrainment to external periodic signals (discussions below). Finally, sustained oscillations are found in a larger range of parameter values for Design II (system robustness, Figures S2 and S3).

### Stimulus Response

Transient stimulation by an external signal can induce oscillations in genetic systems [4,9,11]. These stimulus response dynamics may also be influenced by the different genetic design. We are interested in two scenarios where the stimulus duration and pattern are important. For both cases we suppose that the oscillators are initially in the rest state, i.e., not oscillating, which could be interpreted as the state of no activity of the circuit. The stimulus is able to activate this circuit, pushing it toward the oscillatory regime, e.g., by reducing the repressor degradation.

We first studied the dynamical response to a continuous stimulus with fixed amplitude but varying length. We measured the recovery of the system as the time-lapse needed to return to its rest state after the pulse is switched off. Recovery time increases relatively proportional to pulse duration for short pulses but experiences a sudden threshold for longer ones (Figure S4A). This threshold is due to the presence of a stimulus long enough to trigger an oscillation, and can act as a fail-safe mechanism to avoid unwanted circuit activity, e.g., protecting the cell from high concentrations of oscillator constituent proteins such as p53 [4]. Below this threshold, Design II shows small jumps in response associated to the presence of damped oscillations (Figure S4B).

**Figure 3:**
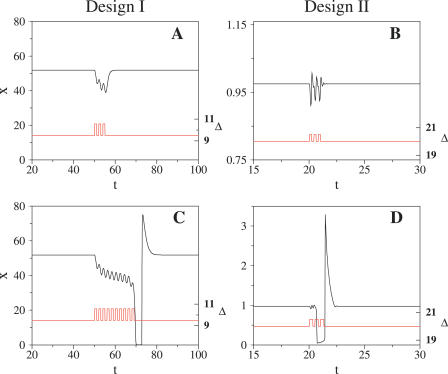
Response in Activator Concentration *(x)* to a Short-Pulse Train (A and C) Design I; (B and D) Design II. (A and B) Three pulses of fixed amplitude and duration. In this example, pulses represent transient changes in repressor degradation rate, i.e., changes in parameter Δ. Pulse durations are chosen approximately as 1/10 of the oscillation period (1 time unit for Design I, and 0.2 time units for Design II, respectively). Pulse amplitudes are 1 for Design I and 0.5 for Design II in units of Δ (right y axis). (C) Design I: ten pulses with the same amplitude and period eliciting a large response. (D) Design II: three pulses with the same amplitude but slightly longer duration (0.3 time units) are able to trigger a big response.

In a second scenario, we analyzed the response of genetic oscillators to different stimulation patterns (Figure 3). We first apply three consecutive signal pulses with the same fixed amplitude, duration, and inter-pulse time to an inactive circuit. This short pulse train does not elicit any significant response in either of the two designs (Figure 3A and 3B). However, a pulse train with the very same features as before, but a larger number of constituent pulses, is “integrated” by Design I which triggers an oscillation (Figure 3C). This is not the case in Design II (figure not shown). We can imagine a complementary situation. In this case, we consider the same pulse pattern as initially but with a slight change in the duration of the pulses (see Figure 3 caption for details). Oscillations are now induced in Design II only (Figure 3D). In summary, Design I acts by integrating external cues, while Design II better detects the periodicity of a given signal. These two different responses have been widely discussed in the context of neurobiology. Neurons in this way can be considered as “integrators” or “resonators,” also as a consequence of the different bifurcation scenarios previously discussed [20,21]. As in neurobiology, and also in the case of calcium signaling [1,19], this has important implications for signal processing.

### Biochemical Noise

Deterministic models present only an approximation to the behavior of genetic oscillators in certain limits, e.g., increasing promoter interactions and number of molecules [24]. In a realistic cellular environment, these systems exhibit stochastic dynamics originated by the presence of a small number of molecules of their constituents [25,26]. Biochemical reactions are then better described in probabilistic terms where the kinetic parameters become transition probabilities. The mathematical description of the system is now in terms of the master equation formalism [27]. The difficulty in solving analytically master equations, even for very simple scenarios, is partially avoided by the use of computational simulations [28].

Stochasticity may play a dual role in genetic systems. Generally, it can impede proper function. As a consequence, some circuit architectures could be preferred to others as being more robust against noise [17,29–31]. Relaxation-based oscillators of the type discussed here have been thought to provide such noise resistance in circadian clocks [17]. However, stochastic dynamics appear as a required feature to perform some biological tasks. This has been recognized in several contexts such as signal amplification [32], noise-induced oscillations [17], and bi-stability [33]. Ultimately, noise can originate phenotypic heterogeneity, which increases the cell adaptation to unexpected environmental conditions [26].

We simulated the stochastic dynamics of the system under different conditions with the Gillespie [28] algorithm (see Supporting Information for details and Figure S7). In all situations, we computed the coefficient of variation (CV, standard deviation/mean) of the distribution of periods and the decay time of the auto-correlation function (τ_*c*_, the time scale at which the periodicity of a dynamical variable is lost) for both designs [27,30] as these are appropriate measures of the variability of oscillations with respect to molecular noise.

**Figure 4:**
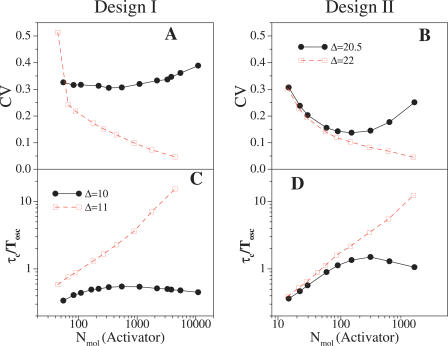
Effect of Noise Due to the Presence of a Small Number of Molecules of All Circuit Components CV and τ_c_ scaled by the deterministic oscillation periods versus biochemical noise expressed as the average number of activator molecules per period, obtained from numerical simulations. (A and C) Design I; (B and D) Design II. Filled circles correspond to a situation close to bifurcation in both designs and open squares to a value of the parameter Δ far from bifurcation. Error bars are the size of data points.

We first studied the effects of noise due to the presence of a small number of molecules of all circuit elements. We modified these numbers by changing a parameter Ω associated with the cell volume. We can envisage two dynamical situations. In the first one, the system is in a non-oscillatory state with the parameter of interest, i.e., Δ, close to the bifurcation value where the oscillations emerge. In Figure 4 it is shown how CV and τ_*c*_ change, in this case as a function of the number of activator molecules. We see how at a certain level of noise CV reaches a minimum value (τ_*c*_ reaches a maximum), i.e., oscillations become more coherent. This is, however, not the regime with the largest amount of molecules of circuit constituents, i.e., the closest to the deterministic noiseless limit, as one could a priori expect. Such apparent paradox is related to a stochastic resonance phenomenon linked to the noise-induced oscillations. This effect is common to both designs, but it is manifested in different ways. In Design I (Figure 4A and 4C), a situation with a large number of molecules reduces the presence of noise-induced oscillations. Oscillations are produced as completely uncorrelated pulses with long waiting times between them, obeying a Poissonian distribution (Figure S5). In the opposite regime of strong noise, oscillations are easily induced but in a very irregular way. The oscillator experiences high period variability (high CV, low τ_*c*_) in both extreme cases.

In Design II (Figure 4B and 4D), the appearance of subthreshold oscillations and a characteristic frequency slightly modifies the previous argument. In this case, a lower minimum in CV is seen due to the inherent higher coherence of oscillations. Variability in the range of weak noise is associated with the presence of different time scales in the system due to the subthreshold oscillations (Figure S5).

We can compare the previous analyses with a far-from-bifurcation scenario (shown also in Figure 4). Stochastic resonance is absent in this case since noise-induced oscillations do not arise. In addition, oscillations in both designs are less variable when the number of present molecules increases, as one would expect. A comparison of Figure 4A and 4B and Figure 4C and 4D reveals a common trend in which Design I dynamics are more variable when the oscillator is close to its bifurcation value.

It is also interesting to analyze the effects associated with low numbers of mRNA species. To examine this contribution to stochasticity, we changed the transcription and translation rates proportionally for a fixed-system volume (Ω). In this way, we are varying the average number of mRNA molecules while keeping the protein levels unchanged. A decrease in transcription, and thus in the number of messenger molecules, is compensated by a “burst” of translational activity [26].

**Figure 5:**
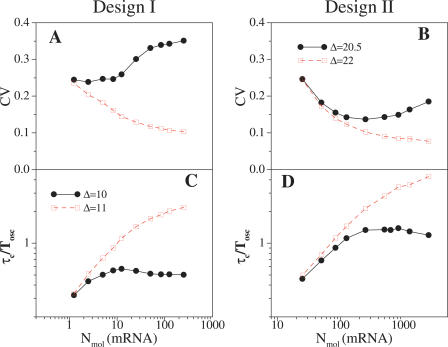
Effect of Noise Due to the Presence of a Small Number of mRNA Molecules CV and τ_c_, as in Figure 4, versus biochemical noise expressed as the average number of activator mRNA molecules per period. (A and C) Design I; (B and D) Design II. Filled circles correspond to a situation close to bifurcation in both designs and open squares to a value of the parameter Δ far from bifurcation. We fix the cell volume such as the system experiences intermediate noise strengths (coinciding with the maxima seen in Figure 4, where the number of activator molecules is ~500 in Design I and ~200 in Design II). Error bars are the size of data points.

In Figure 5 we plotted the variability of oscillations (CVs and τ_*c*_s) as a function of the mRNA molecules of the activator for both designs. We again analyzed two situations with the system within (out) the oscillatory regime. Additionally, we selected a number of molecules of all constituents that correspond to the case of maximal coherence (minimal variability) found in the previous studies (solid curves in Figure 4). These results show that mRNA change contributes strongly to the presence of noise in the system as we obtain similar qualitative behavior by changing mRNA only, than by modifying the number of all molecular species [26]. Transcriptional repression in Design I is reflected in a slightly different behavior of this system with respect to mRNA-induced noise (Figure S6).

### Synchronization

Genetic oscillators are subjected many times to external periodic signals. A common example is that of the circadian rhythms entrained by the external dark-light cycle. More generally, oscillatory stimuli can give important information about natural and synthetic oscillators [34]. Entrainment depends both on the signal and the system-specific features. We are interested in examining how changes in the last properties (i.e., changes in system design) affect entrainment by the same external source.

**Figure 6:**
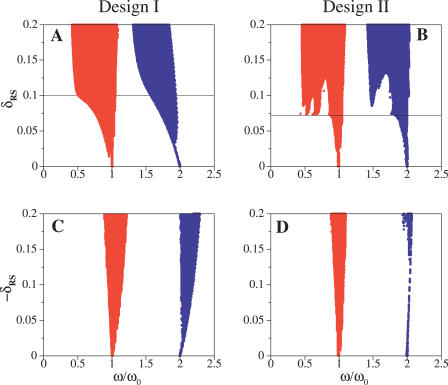
Synchronization Regions (Arnold Tongues) for the Deterministic Models Only the 1:1 (red) and 1:2 (blue) stable resonance regions are shown. ω_0_ is the limit cycle frequency (the undriven system is in the oscillatory regime at Δ = 11 for Design I and Δ = 22 for Design II, respectively), and ω denotes the signal frequency. The (scaled) signal amplitude δ_RS_ affects the repressor degradation (see Supporting Information). Positive values (top panels) increase degradation and decrease the value of Δ while negative values (bottom panels) decrease degradation and increase the value of Δ. Solid lines: critical values of signal amplitude for effectively driving the system towards the rest state.

We considered a periodic square wave signal that modifies the repressor degradation rate when active, e.g., a heat-shock pulse varying the degradation rate of the repressor [16]. This signal can alter the natural period of the genetic oscillator. We analyzed the stable regions of frequency locking, i.e., stable Arnold tongues, as a function of the period and amplitude of the signal for both designs (Figure 6).

A signal increasing repressor degradation can drive the system toward the rest state. Increasing repressor degradation decreases Δ, which needs to be smaller than a given threshold value to quench oscillations. Absence of the signal releases the system back to the oscillatory regime. These combined dynamics ultimately locks the system frequency to that of the external stimulus. Design II exhibits a clearer entrainment threshold in comparison to Design I (Figure 6A and 6B). The lack of a sharp threshold in the first design is a direct consequence of the remnant of the steady-state (ghost state) mentioned in an earlier section. The ghost state induces a slowdown in the dynamics, which allows synchronization with subthreshold signals.

In an alternate scenario, the signal decreases repressor degradation. This effect can only push the system far from the bifurcation accelerating the dynamics. Entrainment by the signal is thus more difficult and the threshold phenomenon disappears. Both systems behave qualitatively in the same way in this case, as we see in Figure 6C and 6D. The slower dynamics of Design I are still reflected in a wider synchronization region.

We studied the effect of biochemical noise in the first entrainment situation (signal-increasing repressor degradation). Noise induces variability in the intrinsic period of the system oscillations and in the phase lag with respect to a reference time, i.e., phase diffusion [30]. In the presence of noise, the effect of a periodic signal is hardly noticed in the CV for the distribution of periods, where only a shift of the maximum of the distribution towards the external signal period is seen for both designs. On the contrary, phase diffusion due to biochemical noise can be counter-balanced by the application of an external signal. This can be appreciated in the decay of the correlations, τ_*c*_, and the distribution of phase lags to the period of the external signal, which can be defined as:


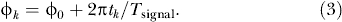


**Figure 7:**
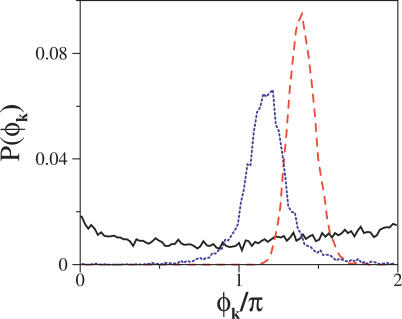
Phase Lag Distributions in the Oscillatory Regime (Δ = 11 in Design I and Δ = 22 in Design II) with biochemical noise at protein levels close to the deterministic limit in both models (the CV of period distribution is ~0.045 in both designs). Solid line (black): no forcing, Design I. Dashed line (red): critical forcing at ω/ω_0_~0.9 in Design I. Dotted line (blue): same parameters for Design II. Design II is more difficult in being synchronized, exhibiting thus more phase diffusion.

Here, *t_k_*, *T*_signal_ are the period of oscillation of the *k*-th cycle and the signal, respectively. In Figure 7 we show the distribution of phase lags for both designs in a situation of weak noise and for a threshold value of the signal. Design II experiences more phase diffusion in agreement with the discussion of the deterministic situation. We also plotted the case of no external forcing for comparison (flat solid line distribution in Figure 7).

## Discussion

The study of minimal architectures capable of generating oscillations is motivated by three complementary aspects. Firstly, simple oscillator motifs seem to be acting as control modules in different biological contexts. This is the case of oscillators involved in the cell cycle of Xenopus laevis [2], the response to DNA damage mediated by the tumor suppressor p53 [4], the establishment of somite patterning in vertebrates [6], or the temporal control of action of the NF-κB transcriptional factor, a basic regulator of many cellular processes [9]. A full understanding of the physiological roles of these oscillators is, however, lacking. Secondly, questions about basic design principles of oscillators such as the relationship among structure, dynamics, and evolution [18], or their modular assembly into complex cellular networks [15], can be more easily analyzed with the use of these simple systems. Finally, a better knowledge of the information-processing capabilities of these schemes is of interest for the bio-engineering of artificial clocks with almost full control of their components and interactions [13,14].

What would one need to know about such minimal architectures to completely understand their function? A usual strategy would be the following: Initially, it is, of course, necessary to identify its molecular components and then characterize their general biochemical properties. Molecular and genetic experiments are later carried out to delineate the logical architecture of its interactions and thus are able to establish the connection between the structure and the function of the module. As a consequence, the identification of similar architectures in different biological contexts could lead us to assume the same functional properties without the demand to characterize experimentally these new situations. In this work, we show the relevance of going from the uncovering of such logical structures, or “molecular cartoons,” to the determination of their specific genetic designs, in order to truly understand their overall functional properties.

We introduced a simple relaxation-based module to analyze the consequences of two alternative genetic implementations on its dynamics. We show that its behavior is dramatically influenced by how the repressor is operating in the system, either sigmoidally (transcriptional repression) or linearly (post-translational repression). These designs are shown to be associated with two different mechanisms originating the onset of oscillations, which in turn determine its dynamical properties.

Both designs differ in basic oscillatory features. Repression operating at the transcriptional level induces oscillations with arbitrarily large periods, while these are smaller and less variable in the post-translational case. In this latter design, the system also exhibits damped oscillatory dynamics. Additionally, in a situation where a similar number of molecules of repressor and mRNA species are present, post-translational repression displays much lower oscillating amplitudes of the activator. These differences greatly influence the functional properties of these designs such as their stimulus response, behavior in the presence of biochemical noise, and entrainment by periodic signals.

Recent experimental reports [4,9,11] have discussed the possibility that certain signaling modules could be encoding information on the amplitude and period of their oscillatory response in order to regulate their transcriptional targets. We show here how these two designs show distinct use of period and amplitudes and also very different signal response to external cues. Design I acts as a signal “integrator” while Design II resonates with specific frequencies of the stimulus (a “resonator”). These behaviors determine the information- processing capabilities of these systems in a similar way to the case of neural systems. Indeed, different neuron types have been shown to respond as integrators or resonators to external signals [21].

Stochastic noise is a relevant factor in understanding the structural and functional properties of genetic oscillators. We studied the behavior of these designs in the presence of two sources of noise, one due to the presence of a small number of molecules of their constituents and the other due to changes in translational efficiency or “translational bursting” [25,26]. Both cases exhibit the well-known phenomenon of stochastic resonance associated with noise-induced oscillations. This means that oscillations in both systems are more coherent for intermediate levels of noise rather than in a weaker noise regime, as one could naively expect. Analyzing both systems under similar noise conditions, we see that Design II exhibits more noise resistance [17]. We also considered a simplistic scenario to study the entrainment of both designs by periodic signals since this is relevant for natural and artificial oscillators [16,34]. Both systems showed distinct entrainment properties. Design I becomes, however, easier to synchronize due to the presence of a ghost state [23].

Understanding the functional consequences of different genetic implementations of minimal motifs emerges as an important requirement to properly classify part of the overwhelming complexity found in cells [35,36].

## Materials and Methods

Numerical simulations were done using MATLAB (The Mathworks, Natick, Massachusetts, United States) and Fortran codes.

## Supporting Information

Figure S1 Bifurcation Diagrams (40 KB EPS)

Figure S2 Robustness Study (71 KB EPS)

Figure S3 Additional Robustness Study (26 KB EPS)

Figure S4 Signal Response (10 KB EPS)

Figure S5 Biochemical Noise Study (118 KB EPS)

Figure S6 Additional Biochemical Noise Study (22 KB EPS)

Figure S7 Time Series of the Reduced/Full Models (230 KB EPS)

Text S1 Deterministic Models Derivation, Parameter Values, and Robustness against Systems Parameters Analyses Signal response and influence of biochemical noise and further discussions. Comparison of reduced and fully developed dynamical models.
(30 KB TEX)

## Abbreviations

CV: coefficient of variation
τ_c_: correlation time
